# Supplementary material for: Hybrid Models and Biological Model Reduction with PyDSTool
Source: PLoS Comput Biol. 2012 Aug 9;8(8):e1002628. doi: 10.1371/journal.pcbi.1002628 (PMC3415397; doi:10.1371/journal.pcbi.1002628)
Supplement: Text S4 — Complete source code for the PyDSTool package (version 0.88.120504). Includes API documentation and help files linking to web pages. This file is identical to the current public release on Sourceforge.net. (ZIP) [file pcbi.1002628.s004.zip › PyDSTool/html/PyDSTool.Toolbox.optimizers.optimizer.standard_optimizer.StandardOptimizer-class.html]

xml version="1.0" encoding="ascii"?


PyDSTool.Toolbox.optimizers.optimizer.standard\_optimizer.StandardOptimizer


| Home | Trees | Indices | Help | PyDSTool |
| --- | --- | --- | --- | --- |

Package PyDSTool ::
Package Toolbox ::
Package optimizers ::
Package optimizer ::
Module standard\_optimizer ::
Class StandardOptimizer

[hide private]

[frames] | no frames]

# Class StandardOptimizer

source code

```
         object --+    
                  |    
optimizer.Optimizer --+
                      |
                     StandardOptimizer
```

---

A standard optimizer, takes a step and finds the best candidate Must
give in self.optimalPoint the optimal point after optimization


|  |  |
| --- | --- |
| Instance Methods | [hide private] |

|  |  |
| --- | --- |
| \_\_init\_\_(self, \*\*kwargs)  Needs to have : | source code |

|  |  |
| --- | --- |
| iterate(self, forceDir=None)  Implementation of the optimization. | source code |

**Inherited from `optimizer.Optimizer`**:
`check_arguments`,
`optimize`,
`recordHistory`

**Inherited from `object`**:
`__delattr__`,
`__getattribute__`,
`__hash__`,
`__new__`,
`__reduce__`,
`__reduce_ex__`,
`__repr__`,
`__setattr__`,
`__str__`


|  |  |
| --- | --- |
| Properties | [hide private] |

**Inherited from `object`**:
`__class__`


|  |  |
| --- | --- |
| Method Details | [hide private] |

|  |  |
| --- | --- |
| \_\_init\_\_(self, \*\*kwargs)  *(Constructor)* | source code |

Needs to have :

- an object function to optimize (function), alternatively a function
  ('fun'), gradient ('gradient'), ...
- a way to get a new point, that is a step (step)
- a criterion to stop the optimization (criterion)
- a starting point (x0)
- a way to find the best point on a line (lineSearch)

Can have :

- a step modifier, a factor to modulate the step (stepSize = 1.)

Overrides: object.\_\_init\_\_

|  |  |
| --- | --- |
| iterate(self, forceDir=None) | source code |

Implementation of the optimization. Does one iteration. (Optional)
Provide known direction to overide step call in 'forceDir'.

Overrides: optimizer.Optimizer.iterate

  


| Home | Trees | Indices | Help | PyDSTool |
| --- | --- | --- | --- | --- |

|  |  |
| --- | --- |
| Generated by Epydoc 3.0.1 on Fri May 4 15:24:09 2012 | http://epydoc.sourceforge.net |
